# CauloKO: an ordered transposon mutant library in *Caulobacter crescentus*

**DOI:** 10.1128/jb.00417-22

**Published:** 2026-02-27

**Authors:** Gabriel M. Moore, Justin G. Ramos, Joseph P. Sheehan, Benjamin P. Bratton, Zemer Gitai

**Affiliations:** 1 Department of Molecular Biology, Princeton University 200547, Princeton, New Jersey, USA; 2 Department of Biology, Oberlin College 6167, Oberlin, Ohio, USA; 3 Department of Pathology, Microbiology and Immunology, Vanderbilt University Medical Center 204907, Nashville, Tennessee, USA; University of Notre Dame, Notre Dame, Indiana, USA

**Keywords:** genetic library, *Caulobacter crescentus*, CauloKO, biofilms, holdfast, transposons

## Abstract

Genetic screens are powerful approaches to unveiling new biological insights and ordered redundant transposon libraries have emerged as a primary tool for performing screens with known genetic saturation. Newer sequencing approaches based on combinatorial pooling have lowered the cost and time required to generate these libraries. *Caulobacter crescentus* is a gram-negative bacterium that has served as a model for understanding bacterial physiology with a myriad of genetic tools. To add to this collection of tools, we created CauloKO—the first ordered, transposon library in *C. crescentus*. CauloKO includes insertion mutants in 86% of all non-essential genes and 77% of all open reading frames of strain CB15. CauloKO insertion mutants were validated using Sanger sequencing. We also present phenotypic analysis of the CauloKO library using a crystal violet screen for biofilm mutants, which both confirmed previous results and identified new mutants for future studies. This combined approach revealed that the CauloKO library shows promise for screening applications, particularly for phenotypes that require monoclonal populations of cells.

**IMPORTANCE:**

Ordered transposon mutant libraries have dramatically advanced the ability to study other systems, and the potential for combining such a library with the other genome-wide tools available in *Caulobacter crescentus* makes such a library particularly important for this system. Our work also establishes a novel method for analyzing sequencing data for ordered library construction purposes, and a first-of-its-kind dynamic, online platform for cataloging mutants within the library. Furthermore, our findings further confirmed results of previously published studies, but also identified potential novel regulators of biofilm formation in *C. crescentus* that will be important starting points for future investigations.

## INTRODUCTION

Our understanding of microbiological processes heavily relies on genetic screens. Transposon screens are particularly powerful because transposons typically result in loss-of-function mutations and mapping their insertion sites is fast and easy. Classical genetic screens introduce random transposon insertions to a population of cells and then examine the resulting colonies for a particular phenotype, or lack thereof (1). While this method has the advantage of being quick and easy for identifying individual mutations, Poisson statistics make it difficult to achieve saturation and ensure every open reading frame (ORF) is disrupted (2). On the other end of the spectrum, high-density TnSeq approaches are a powerful way to study the entirety of the functional genome (3). However, TnSeq studies are performed in complex mixed communities such that they cannot be applied to clonal populations, and it is often difficult to retrieve specific transposon insertions for further studies. These limitations have been overcome by ordered transposon libraries that attempt to include clonal isolates of as many mutants as possible that can tolerate a transposon insertion (4–9). Several clinically relevant bacterial species have had libraries constructed in at least one strain (4–9), but the significant cost and effort typically required to generate these libraries has limited their widespread application.

Recently, strategies such as Knockout Sudoku and Cartesian Pooling-Coordinate Sequencing (CP-CSeq) have been developed for rapid determination of the identity and location of transposon mutants in a library with minimal resources (10–13). These strategies rely on arraying transposon mutants in 96-well plates into matrices that give each mutant a set of coordinates that can be determined in custom sequencing pipelines. These methods of combinatorial pooling and decoding of transposon mutants have led to an increase in the number of ordered transposon libraries available, including in *Mycobacterium bovis*, *Shewanella oneidensis*, and *Bacteroides thetaiotaomicron* (12–14).

*Caulobacter crescentus* is a gram-negative, alpha-proteobacterium predominantly isolated and associated with aqueous and rhizosphere environments (15–17). *C. crescentus* has emerged as a powerful model system for a wide range of important characteristics including its canonical “crescent” cell shape, cell cycle, and polarity (17–19). Recent studies have also suggested that *C. crescentus* can function as an animal pathogen (20). While many of these processes have been studied using classical genetic techniques, the continued discovery of novel determinants in *C. crescentus* is aided by a number of tools generated in the organism, including a cosmid library (21), a fluorescently-tagged localisome library of ORFs (22), and a previously available web-based visualizer of all published high-throughput genomic data (23). At present, however, there is no ordered transposon library to facilitate genetic screens that require monoclonal populations of cells or to provide a resource of mutants-of-interest for genetic analysis.

Given the many resources that already exist in *C. crescentus* and relative ease of library generation with modern sequencing methods, we sought to create and assemble an ordered transposon library in *C. crescentus*. We used a combination of tools from the Knockout Sudoku pipeline and a homebrewed sequencing analysis pipeline to generate CauloKO, a library with 77% coverage of predicted ORFs and 86% coverage of non-essential genes in the *C. crescentus* genome. To validate the usefulness of CauloKO as a tool, we phenotypically characterized this library by using a crystal violet screen. We identified biofilm mutants that were both consistent with past work and highlighted novel candidates for further exploration. Our work thus adds to the wealth of tools available for understanding *Caulobacter* physiology in particular and bacterial biology in general.

## RESULTS AND DISCUSSION

### Transposon mutagenesis and construction of initial mutant collection

**Fig 1:**
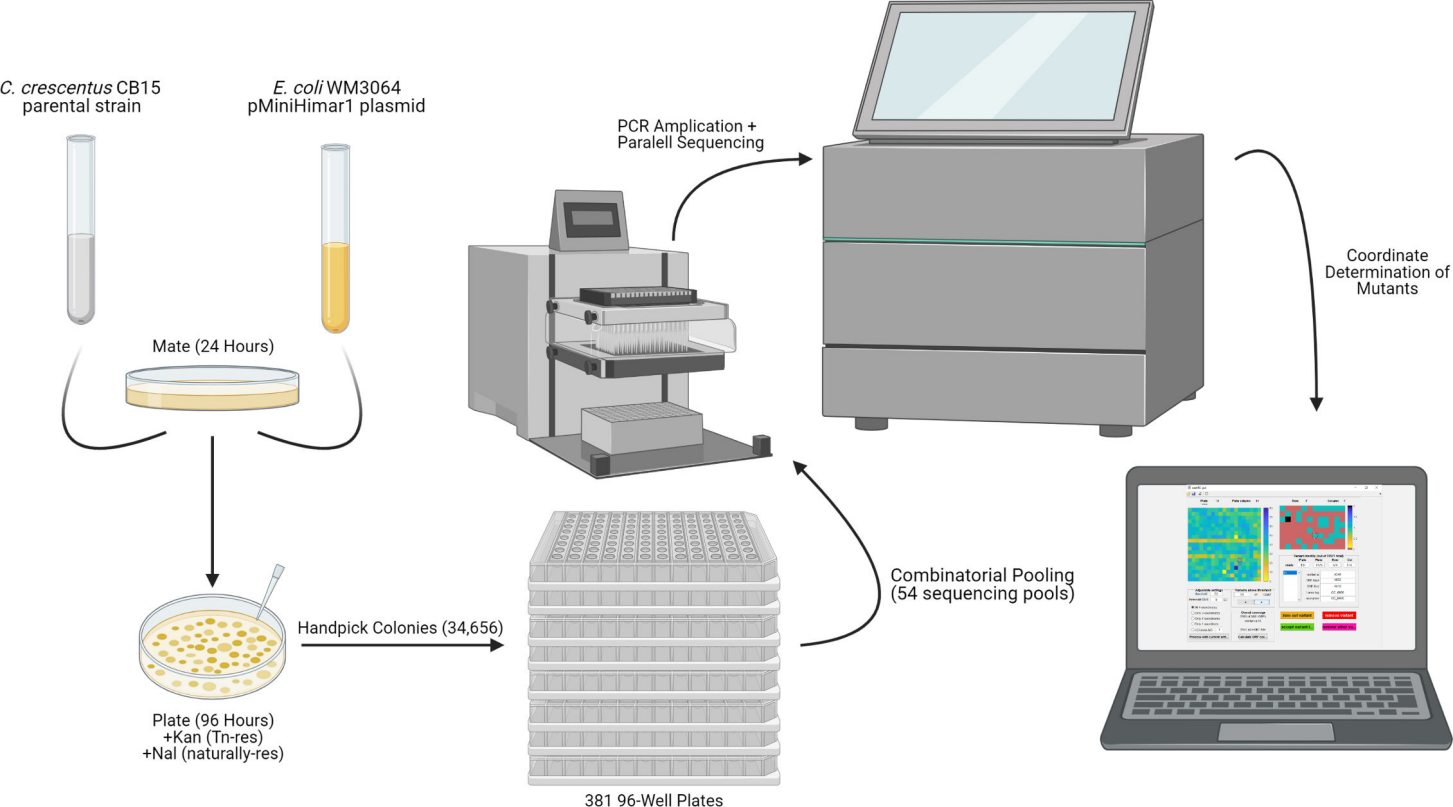
Methodology for physical construction of the *C. crescentus* CB15 CauloKO library. *C. crescentus* CB15 cells were mated with pMiniHimar1 containing *E. coli* WM3604, and resulting colonies were handpicked in 96-well plates. Plates were pooled into 54 sequencing pots that were barcoded and PCR amplified before sequencing. The coordinates for sequenced mutants were then determined using the CauloKO GUI generated in this study.

We chose to use the environmentally isolated *C. crescentus* CB15 background as the parental strain for our ordered library, as it retains holdfasts and other phenotypes that have been lost in lab-adapted strains (24). To generate transposon insertions, we mated *C. crescentus* CB15 with pMiniHimar1-possessing *Escherichia coli* WM3064 and selected colonies by plating on antibiotic agar plates with kanamycin and nalidixic acid (Fig. 1). Kanamycin resistance selects for the presence of the miniHimar1 transposon, and *C. crescentus* is naturally resistant to nalidixic acid, thereby eliminating *E. coli* contamination (25).

The next step was to pick a large number of *C. crescentus* transposon-containing colonies with the goal of saturating the CB15 genome. CB15 contains 3,767 predicted ORFs. While we have essentiality data for many of these ORFs based on high-density TnSeq analysis of the lab *C. crescentus* strain, NA1000 (26), we calculated an upper estimate for the number of transposon mutants we should include by assuming that all genes have the potential to tolerate transposon mutagenesis. A Monte Carlo-based simulation predicted that 34,656 transposon mutants would cover 99% of all ORFs (Fig. 1; Fig. S1). We note that the Monte Carlo estimate is superior to a Poisson estimate for saturation as it takes into account the length of each gene, so as not to overestimate insertions in long genes nor underestimate insertions in shorter genes (13). We thus handpicked 34,656 kanamycin-resistant CB15 colonies from our pMiniHimar1 mating into 361 multi-well plates, each with 96-well plates.

We next used the Knockout Sudoku approach to pool our 361 plates into sequencing pools (13). In brief, the 361 plates were organized into a 19 × 19 higher-order array. From this array, we generated 54 mutant pools, including 12 “column” pools and 8 “row” pools (pooling all of the mutants from each column and row across all 361 plates), as well as 19 “plate column” pools and 19 “plate row” pools (pooling all of the mutants from the entire 96-well plates in each of the 19 × 19 higher-order plate-columns and plate-rows). These 54 pools enable us to identify each specific mutant from only four coordinates: columns of the plates (1–12), rows of the plates (A–H), and the plate column and plate row within the 19 × 19 array. Each pool was PCR-amplified, barcoded, and sequenced by Illumina Sequencing.

To ensure no major genomic differences occurred in the time it took to pick, pool, and move all mutants to frozen storage, we performed full genome sequencing of the parental strain and four random transposon mutants from the collection. A total of 46 SNPs were identified in the parental and mutant strains compared to the reference genome for *C. crescentus* CB15 (Table S1) (27). All SNPs identified in the parental strain were shared with at least one of the transposon mutants. The majority of these SNPs were in intergenic regions or represented silent mutations, and thus unlikely to have an effect on gene products. All transposon mutant strains possessed between one and three additional SNPs not found in the parent strain, with unknown impacts on gene products if not intergenic. The majority of these non-congruent SNPs were similarly in intergenic regions or had minimal effect on the genetic identity of the mutant. Furthermore, many of these SNPs were shared between transposon mutants and may represent SNPs present yet not identified in the parental strain due to sequencing coverage. Thus, the parental strain for library construction reflects a representative CB15 strain and shares nearly identical genomic similarity to the transposon mutants aside from their respective transposon insertion (Table S1). To see how these changes compare to the NA1000 genome, previous work has compared the gene loci of the two strains (27).

### Sequencing analysis of the initial mutant collection and assembly of the ordered CauloKO library

**Fig 2:**
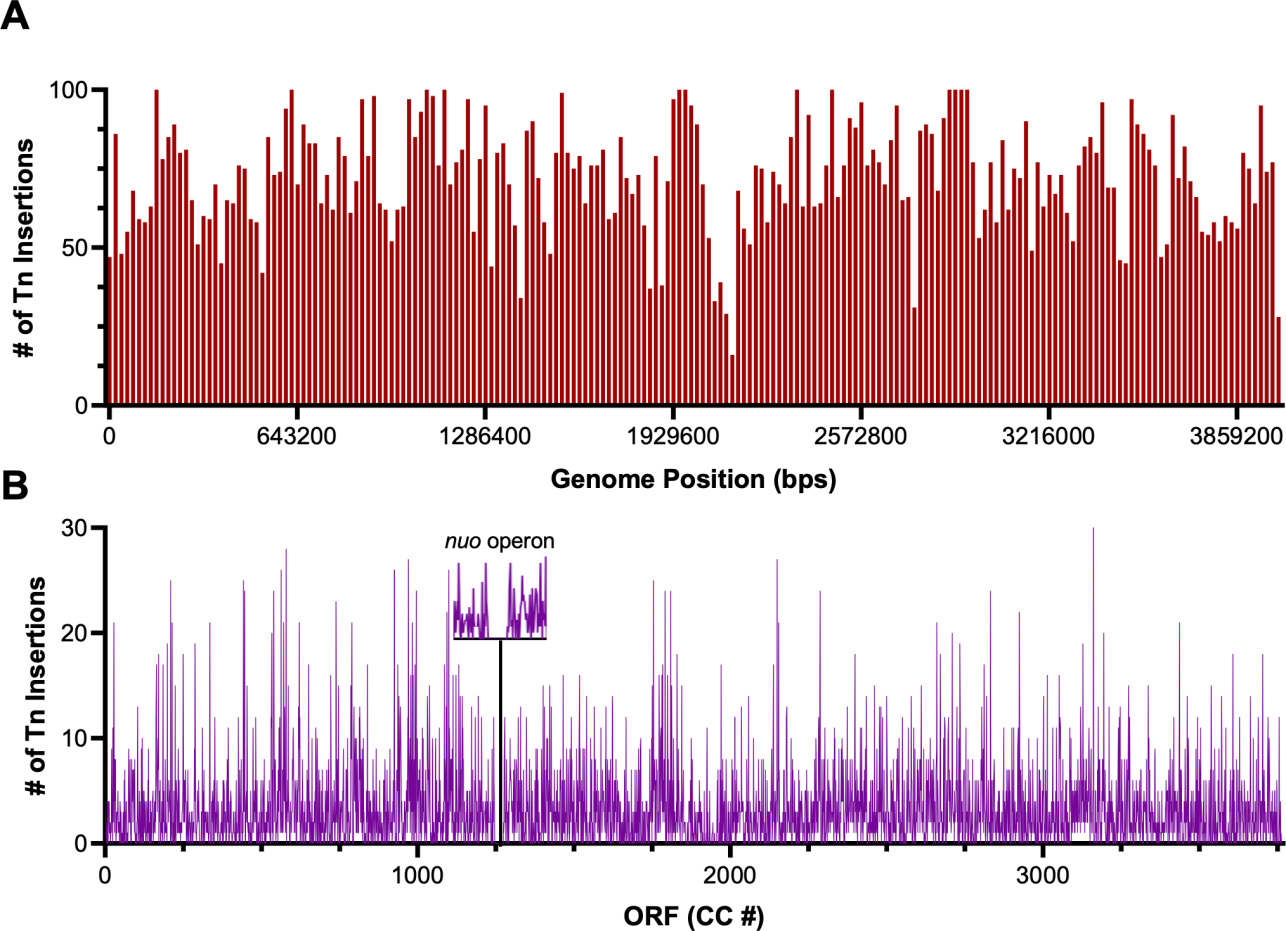
Unique transposon insertion saturation by (**A**) genome position and (**B**) open reading frame (ORF). Histogram in (**A**) consists of 200 bins with each bin = 20,000 bps. Gap in sequencing reads of transposon insertions indicative of the essential *nuo* operon.

Sequence analysis of the transposon library pools revealed that our library achieved broad coverage of the *C. crescentus* genome. Due to the pooling and barcoding methodology for sequencing, not all 34,656 mutants were able to be captured as many mutants did not pass the quality thresholds after barcoding and sequencing. However, we successfully identified 22,219 unique insertions across the CB15 genome via sequencing, with an insertion rate of ~1 transposon per 278 base pairs (bps). As expected, operons that have previously been shown to be essential were under-represented, as in the case of the essential *nuo* operon for NADH dehydrogenase (CC_1950–CC_1956), whose insertion rate was 1 transposon per 1,250 bps (Fig. 2) (28). Similarly, we observed no insertions in the essential ribosome subunit operon found from CC_1245 to CC_1273. Overall, the initial collection of 22,219 insertions included at least one transposon insertion in 85.6% of all predicted genes.

To generate the CauloKO ordered insertion library, we proceeded to use the Knockout Sudoku approach to determine the plate locations of specific insertion mutants in the initial collection. While the previously published Knockout Sudoku pipeline was instrumental in constructing the initial collection and performing combinatorial pooling (13), the signal-to-noise ratio (SNR) in our sequencing data could not be tolerated by the Bayesian inference used in the published Knockout Sudoku software. Thus, we developed our own software to deconvolve the pooled sequencing results. Specifically, we generated a MATLAB GUI that enabled us to semi-automatically determine the predicted coordinates of our mutants based on primary and secondary thresholding requirements of the sequencing read counts (Fig. S2). The primary threshold was filtered by the absolute number of read counts for a given coordinate. Secondary thresholding determined the SNR of read counts by comparing the highest value for a particular coordinate to the next highest value, as the highest number of read counts was typically the most accurate coordinate of the mutant. Automated filtering by absolute read counts and SNRs provided a high-confidence set of mutants with known locations based on their coordinates. Determining the identity of these mutants with high confidence also enabled us to manually eliminate their positions as possible locations for other mutants, thereby enabling us to triangulate the positions of additional mutants with fewer known coordinates from the initial analysis. The combination of automated and manual mutant selection using the CauloKO GUI enabled us to determine unique genomic insertion sites and plate coordinates for 20,345 mutants, or about ~70% of total mutants picked (Table 1).

**TABLE 1:** Essentiality of open reading frames (ORFs) contained in the sequencing and CauloKO library

	ORFs hit	ORFs missing	Total
Sequencing data			
Non-essential (%)	2,731 (87.1%)	404 (12.8%)	3,135
High fitness (%)	118 (75.6%)	38 (24.4%)	156
Essential (%)	376 (79.0%)	100 (21.0%)	476
Total (%)	3,225 (85.6%)	572 (14.4%)	3,767
CauloKO library			
Non-essential (%)	2,687 (85.7%)	448 (14.3%)	3,135
High fitness (%)	90 (57.7%)	66 (42.3%)	156
Essential (%)	125 (26.3%)	354 (73.7%)	476
Total (%)	2,902 (77.0%)	865 (23.0%)	3,767

### CauloKO provides broad coverage of the non-essential genome of *C. crescentus* CB15

**Fig 3:**
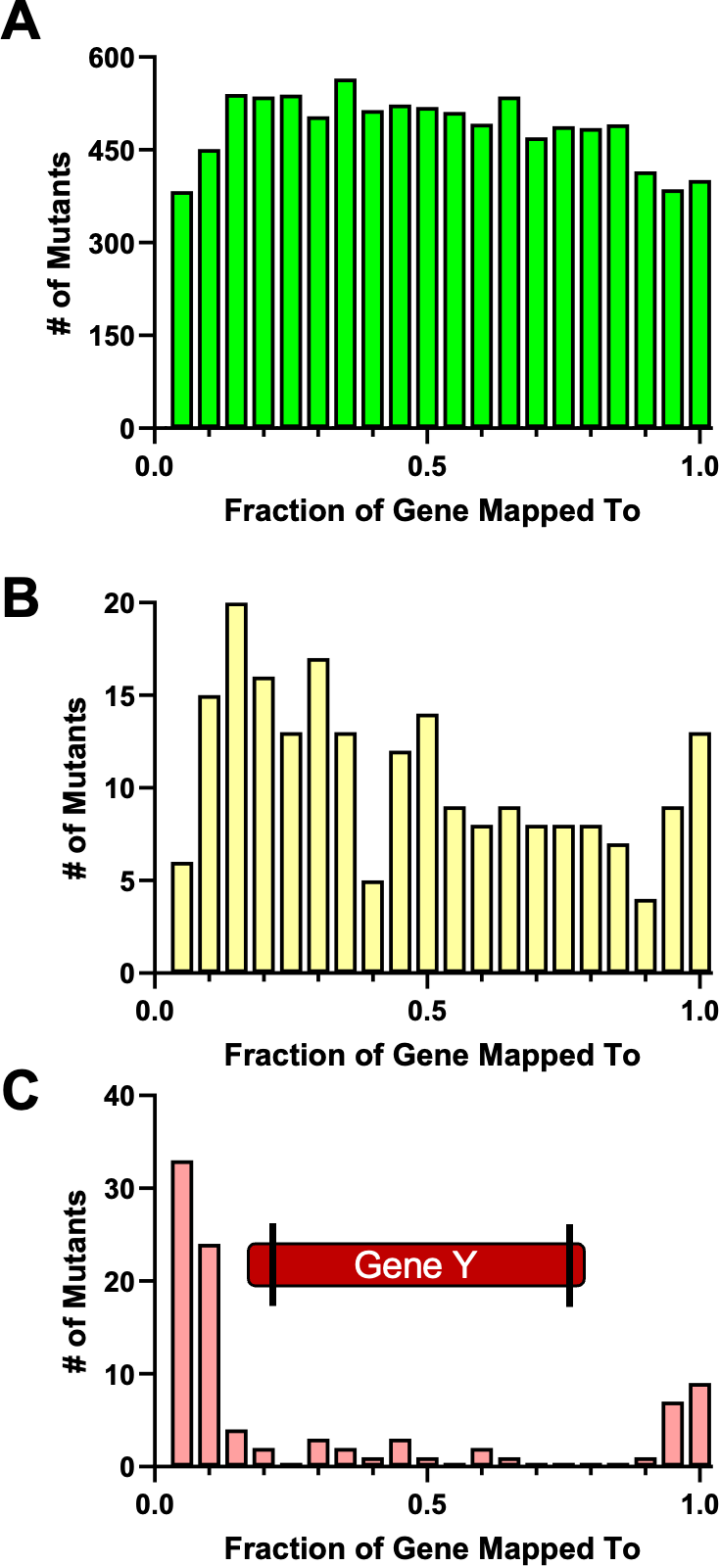
Mapping of transposon insertions to position of genes in CauloKO library. (**A**) Non-essential genes (*n* = 9,749). (**B**) High fitness genes (*n* = 214). (**C**) Essential genes (*n* = 96). Each bin represents 0.05 fractions of the gene. A diagram of “gene Y” indicates the locations of the transposon insertions within the gene for essential genes.

The 20,345 mutants whose positions in the initial collection could be uniquely identified weere mapped to 2,902 genes that we consolidated into an ordered CauloKO library. CauloKO represents 77% of all predicted ORFs, including 86% of genes previously annotated as non-essential (26), 18% of genes previously annotated as essential (26), and 19% of “high fitness” genes (genes previously shown to tolerate transposon mutagenesis at lower rates than other non-essential genes [26]) (Table 1). We hypothesized that the presence of insertions in essential and high fitness genes in our library was due to either strain-specific differences in essentiality between CB15 and NA1000, or retention of partial gene function upon transposon mutagenesis. Retention of partial gene function would be expected for insertions at the ends of ORFs (where enough gene product is retained to maintain cell viability) or the beginnings of ORFs (where alternative start sites or readthrough from transposon promoters could maintain expression of most of the gene). To test this hypothesis, we mapped transposon insertion sites relative to the fraction of the gene into which each transposon inserted. As expected, we found that insertion sites in non-essential genes were uniformly distributed throughout the genes, while insertions in essential genes were biased to the gene ends (Fig. 3). Altogether, only six genes previously annotated as essential contained insertions in the middle of the gene. Given this low number and the fact that some of the six genes with central insertions might be essential in NA1000 but not CB15, our results suggest that our library’s coverage and annotation accuracy are robust.

### Genetic verification of select mutants and CauloKO portal for community annotation

To confirm the accuracy of our transposon mutant annotations, we used random priming to independently sequence the insertion sites of 53 randomly selected transposon mutants from the CauloKO library. Our results indicated that 40 of the 53 mutants (75%) contained insertions in either the predicted gene or the gene immediately adjacent (Table S2). Past efforts have noted that transposon library creation leads to a fraction of inaccurately annotated mutants, and this misannotation rate is not known for many libraries (3–7). This problem has been addressed by creating redundant library collections when possible—with a 75% accuracy rate, the chance that at least one of two insertions in a given gene is correct is 94% and for genes with three insertions, that chance rises to 99%. Thus, we included mutants in triplicate when possible in the final collection, leading to the final CauloKO library of 6,739 mutants from the 20,345 mutants which we were able to identify and list in Table S3. In this final collection, 54% of the ORFs were represented by three independent insertions, 25% by two insertions, and only 21% by one insertion (Table 2). Taken together, we expect that 93% of the ORFs in this collection are accurately represented by at least one insertion in the corresponding gene. We also included several mutants with insertions in intergenic genomic regions as internal controls, as these do not create disruptions to predicted gene products but do contain a transposon (Table S3). For ease of screening more complex phenotypes, we also created a “consolidated” collection of the library containing 2,902 mutants with a single mutant for each available ORF, with the representative mutant having a transposon inserted at the most upstream possible position to favor disruption of the gene product (Table S4).

**TABLE 2:** Number of insertion mutants and genetic mutants present in single, duplicate, or triplicate in CauloKO

Redundancy	# of Mutants
Single	625
Double	717
Triple	1,560
Total	6,739
Intergenic	65

No library is perfect and future work by the community can help to significantly improve CauloKO and its annotation. Consequently, we developed a mechanism for members of the community that use CauloKO to update the rest of the community in real-time as to the identity and properties of mutants in the collection. This is a problem with other ordered libraries, as annotations are rarely updated when passed to a new user. Specifically, we created an online portal to allow for individuals from any lab to add updated gene annotations for the CauloKO library from Sanger sequencing or other sequencing methods to confirm annotations (https://www.gaybelab.com/cauloko).

### Phenotypic validation by crystal violet screening

Using the consolidated collection, we wanted to demonstrate the utility of the CauloKO library in a phenotypic screen that would both validate known genes and identify novel genes with a particular phenotype. For this purpose, we assayed our library for biofilm formation using crystal violet staining for quantification (29–31). While surface attachment has been robustly examined in *C. crescentus* and is known to be involved in biofilm formation (32), crystal violet screening has not been previously performed in *C. crescentus*. Thus, known surface attachment mutants should be identified by our screen as positive controls. Furthermore, biofilm formation is a population-level phenotype such that this assay highlights the power of screening our library in clonal populations.

Surface attachment of *C. crescentus* is mediated by motile swarmer cells that use type IV pili (TFP) for initial adhesion. Retraction of the pilus initiates cell cycle progression, transforming swarmer cells into surface-adherent stalked cells (33–35). This irreversible attachment is mediated by the holdfast, an incredibly strong adhesin that is synthesized intracellularly, exported to extracellular space, and anchored to the end of the stalk (36–39). Once attached, microcolony formation and biomass production lead to larger-scale biofilm structures. Holdfast synthesis and anchoring, pilus activity, and flagella presence are required to promote higher-order biofilm formation (32). Additionally, extracellular DNA from *C. crescentus* can inhibit biofilm formation by blocking holdfast attachment to surfaces (40). While each of these processes has been suggested to be involved in biofilm formation, the lack of previous systematic biofilm screens leaves unclear the extent to which each of *C. crescentus’* attachment modalities and other processes contribute to biofilm development.

**Fig 4:**
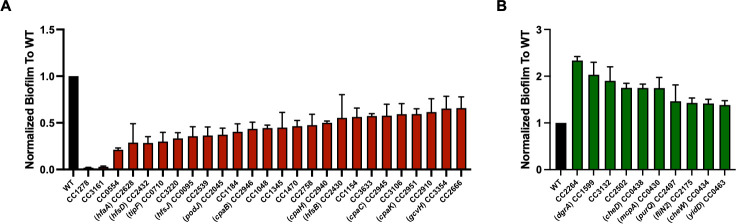
Quantification of significant mutants from crystal violet screen. (**A**) Mutants decreased for biofilm formation by crystal violet staining. (**B**) Mutants increased for biofilm formation by crystal violet staining. All mutant strains shown in this figure are significantly different from the control (*P* < 0.05, unpaired *t* test).

**Fig 5:**
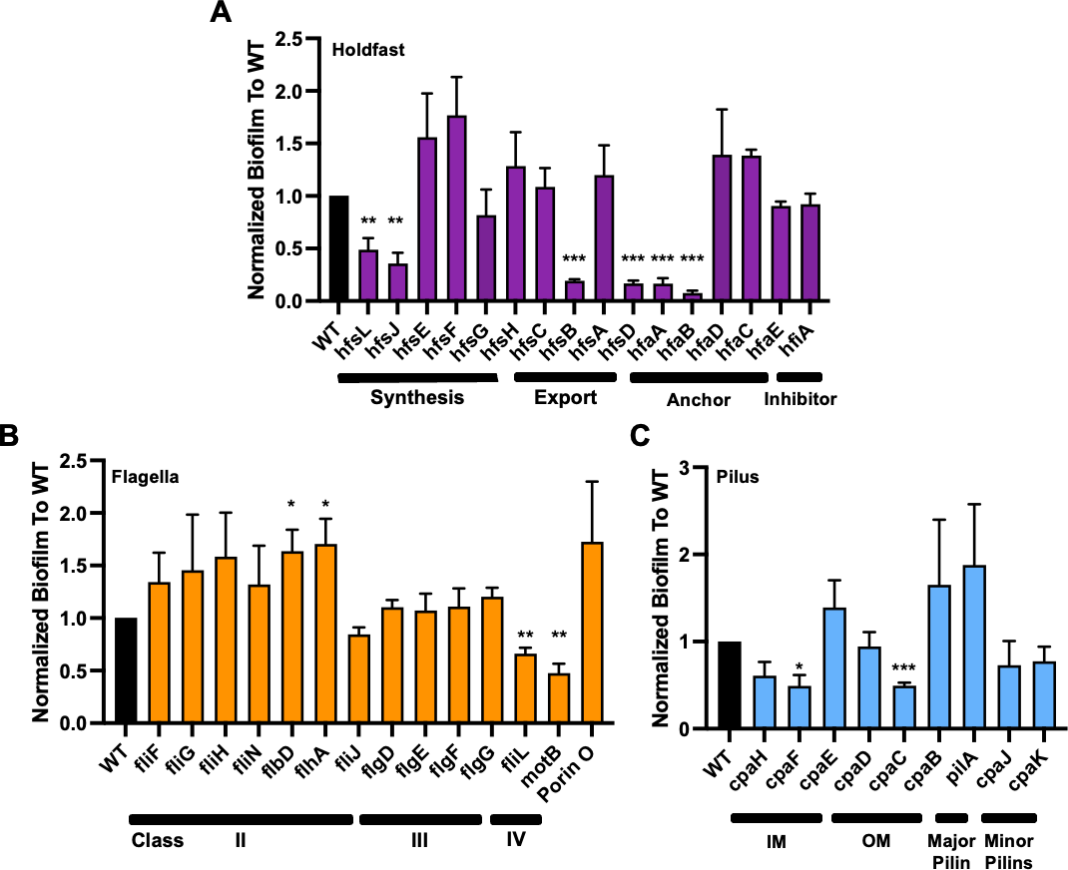
Select mutants related to biofilm formation that replicate known phenotypes. Holdfast (A, purple), flagella (B, orange), and pilus (C, blue) mutants were measured for crystal violet staining normalized to biomass after forty-eight hours of growth. (**A**) Holdfast mutants examined are involved in the synthesis of holdfast, export of holdfast to the stalk, anchoring of holdfast to the stalk, or inhibition of the process of holdfast synthesis during the cell cycle. (**B**) Flagellar mutants examined are represented by Class II, III, and IV flagellar genes and those present within the same operon as Class II genes (e.g., porin O). (**C**) Pilus mutants examined represent components associated with the inner membrane (IM) and outer membrane (OM) as well as major and minor pilin subunits. Error bars represent standard error of the mean. *cpaG* and *cpaA* of the type IV pilus operon are not present in the CauloKO library. Statistical significance of mutant strains was determined by unpaired *t* test compared to the control (*P < 0.05, **P < 0.01, ***P < 0.001).

To establish the power of CauloKO for genetic screens and phenotypic analysis of mutants of interest, we screened the consolidated CauloKO library for biofilm formation by measuring normalized crystal violet staining after 48 h of growth in static 96-well plates. Plates were stained, measured for crystal violet (by OD_540_), and normalized for bacterial growth (by OD_660_) to determine biofilm presence in biological triplicate. Mutants with 1.5-fold or greater decreased/increased biofilm formation relative to the average biofilm formation of their respective plate are listed in Table 3. Of the mutants identified in our screen, we more rigorously quantified the crystal violet staining observed in these mutants relative to wild-type by growing overnight cultures instead of directly from the library plates to use in this assay and determine the statistical significance of our initial screen (Fig. 4). Additionally, because biofilm formation has previously been linked to genes associated with holdfast, flagella, and tTFP, we further evaluated biofilm formation in a selection of CauloKO mutants with insertions in genes related to these pathways (Fig. 5). All mutants assessed in this manner were pulled from the consolidated version of the CauloKO library regardless of their identification in our original screen (Table 3 and Fig. 4). This second approach also served as validation of previously studied genes that contribute to biofilm formation and aided in determining if these mutants behaved similarly in our assay.

**TABLE 3:** Mutants identified in crystal violet screen of CauloKO library

ORF # (CC)	Predicted function
Decreased biofilm formation	
95	WecG/TagA-family glycosyltransferase hfsJ
133	Ketoacyl reductase hetN
136	Uncharacterized protein
247	Two-component receiver protein spdR
451	Zn-dependent hydrolase, glyoxalase II family
554	Uncharacterized protein
710	Cyclic-di-GMP phosphodiesterase, flagellum assembly factor tipF
853	Uncharacterized protein
898	Flagellin flgL
998	ABC transporter ATP-binding protein
1048	Acylamino-acid-releasing enzyme
1080	LuxR-family transcriptional regulator
1096	Ribosomal RNA large subunit methyltransferase J
1154	dATP pyrophosphohydrolase
1184	Amino acid permease
1278	GMC family oxidoreductase
1345	TetR/AcrR family transcriptional regulator
1372	Uncharacterized protein
1403	Cytochrome c oxidase, ccoQ subunit
1470	Acetyltransferase, GNAT family
1486	WbqP Undecaprenyl-phosphate beta-N-acetyl-D-fucosaminephosphotransferase
1641	2′−5′ RNA ligase family protein
1732	Ferritin-like domain protein
1737	Competence/damage-inducible protein cinA
1906	Citrate synthase
2045	Localization factor PodJL
2253	Lysophospholipase L2
2385	Uncharacterized protein
2430	Polysaccharide autokinase-related protein hfsB
2432	Polysaccharide secretin protein hfsD
2466	DNA polymerase IV dinB
2539	M61 glycyl aminopeptidase
2601	Cyclopropane fatty acid synthase-like protein
2628	Holdfast attachment protein hfaA
2629	Putative transcription activator protein hfaB
2642	Choline dehydrogenase
2666	Uncharacterized
2758	Periplasmic serine endoprotease DegP-like
2910	Nucleoside/nucleotide kinase
2928	TonB-dependent receptor
2940	TadC-related pilus assembly protein cpaH
2945	Pilus assembly protein cpaC
2946	Pilus assembly protein cpaB
2951	TadE-related pilus assembly protein cpaK
3106	Alanine racemase
3161	TonB-dependent receptor
3217	Uncharacterized protein
3220	Uncharacterized protein
3266	RNA polymerase sigma factor, SigJ family
3380	2-deoxy-d-gluconate 3-dehydrogenase
3412	Acetyltransferase, GNAT family
3572	Carbonic anhydrase
3633	dTDP-4-dehydrorhamnose 3,5-epimerase
3639	FtsZ-localized protein A fzlA
3705	Outer-membrane lipoprotein carrier protein
Increased biofilm formation	
1184	Amino acid permease
430	Chemoreceptor mcpA
434	Chemotaxis protein cheW
435	Chemotaxis protein methyltransferase
436	Protein-glutamate methylesterase/protein-glutamine glutaminase 1 cheB1
438	Probable chemoreceptor glutamine deamidase cheD
463	Putative membrane protein insertion efficiency factor
663	TonB-dependent receptor
752	Hypoxia transcriptional regulator fixK
863	Uncharacterized protein
993	Conserved hypothetical cytosolic protein
1599	Diguanylate receptor protein dgrA
1661	Alanine racemase
1700	Uncharacterized
1744	d-alanine aminotransferase
2175	FliN family protein
2264	Phosphomannomutase/phosphoglucomutase
2497	Phosphoribosylformylglycinamidine synthase subunit purQ
2502	Aminopeptidase
2520	SpoU rRNA methylase family protein
2575	3-hydroxybutyryl-CoA dehydratase
2604	Serine-pyruvate aminotransferase
3027	Conserved membrane spanning protein
3083	NADH-dependent flavin oxidoreductase
3132	Acetyl ornithine aminotransferase
3195	Outer membrane protein oprM
3217	Uncharacterized protein
3237	Holliday junction ATP-dependent DNA helicase ruvA
3274	Multidrug/protein/lipid ABC transporter family, ATP-binding and permease protein
3346	Esterase/lipase
3482	SWIM zinc finger family protein

We first determined whether the mutants we identified in either our screen or our specified genes were consistent with previous findings and found that our data largely agreed with the results that would be expected based on previous studies. For example, chemotaxis mutants, such as *cheD* and *cheW*, are known to stimulate biofilm formation, while the stalk development mutant, *podJ*, is known to reduce biofilm formation, and we found that these mutants affected crystal violet staining in the expected direction in our screen (Fig. 4) (41, 42). Holdfast synthesis was also previously shown to be required for crystal violet staining in exponentially growing cells. Consistently, most genes annotated as essential for holdfast formation in the exponential phase also displayed reduced crystal violet staining in our screen (Fig. 5A). Some holdfast-related genes are regulatory or are redundant and thus did not eliminate holdfast synthesis itself, such as *hfaD* and *hfsC* (36, 37). In agreement with prior studies, these holdfast-associated and redundant mutants that do not reduce the formation of the holdfast itself did not decrease crystal violet staining (Fig. 5A) (37, 39). Across all holdfast-related genes, we found only two whose results from our stationary-phase crystal violet screen did not agree with previous reports from exponential phase studies: glycosyltransferase *hfsG* and holdfast synthesis protein *hfsA* (Fig. 5A) (36–39). These discrepancies could be due to misannotation of these transposon mutants, an insertion of the transposon at the end of the gene that does not disrupt gene function, or differences in the specific conditions used for the different assays (32). Based on transposon insertion data, we have determined that *hfsG* and *hfsA* possess transposon insertions toward the end of the gene, which would indicate a lack of phenotypic disruption or semi-functionality of the protein product. Determination of transposon insertions of these genes and all other mutants of interest can be found in the consolidated collection (Table S4). Additionally, our assay examined stationary phase cells in a non-shaking condition, which differ from exponentially growing cells under shaking conditions, potentially mimicking more environmentally relevant conditions where biofilm formation occurs.

While the role of the holdfast in crystal violet staining is clear, the role of flagella and TFP in biofilm formation is more complicated. Early work in *C. crescentus* biofilms demonstrated that flagellar presence but not function was necessary for forming higher-order biofilm structures (32). More recent work demonstrated that flagellar disruption stimulates holdfast synthesis, which would be predicted to stimulate crystal violet staining in our screen (43, 44). Looking specifically at Class II and III flagellar and motor genes, we observed two mutants with decreased biofilm formation in our screen and several flagellar mutants with increased biofilm formation (Fig. 4B and 5C). In particular, the CauloKO *motB* mutant phenocopied the decrease in biofilm production as described previously for a *motB* deletion (38). *fliL* also demonstrates lower crystal violet staining, suggesting that mutants where a flagellum is present but not functional reduce biofilm formation. *flbD* and *flhA* exhibited significantly higher crystal violet staining, as did other Class II flagellar genes (with broader distributions). As described previously, this may suggest that lack of flagellar presence stimulates biofilm formation (43). However, the majority of flagellar mutants retained wild-type levels of biofilm formation, suggesting that there may be redundancy or specific roles for individual flagellar components in holdfast regulation and biofilm formation.

TFP is an extracellular polymer in which PilA pilin monomers are assembled and disassembled by a TFP motor (33). As with our holdfast and flagellar interrogation of crystal violet staining, many of our results phenocopied that of previously established literature, and other results pointed to interesting new directions for future studies. For example, we found that loss of the *pilA* TFP subunit increased biofilm formation and loss of TFP motor mutants decreased biofilm formation despite the fact that these factors are generally thought to function together (Fig. 4A and 5A). Interestingly, a recent TnSeq study of *C. crescentus* attachment to cheesecloth also found opposite attachment phenotypes for *pilA* and TFP motor mutants, suggesting that unassembled PilA monomers regulate holdfast formation (45). Thus, while further studies will be needed to dissect the regulatory cross-talk and timing of how flagella, TFP, and holdfast structures affect one another and biofilm formation, our results both confirm previously described regulatory interactions and identify new questions for future work.

Our screening results also identified potential novel players involved in *C. crescentus* biofilm formation (Fig. 4). Among mutants with decreased crystal violet staining, two mutants stood out as having remarkably low holdfast staining: CC_1278, which encodes a predicted GMC family oxidoreductase, and CC_3161, which encodes a TonB-dependent receptor. These putative gene functions have not been previously identified as necessary for biofilm formation in *Caulobacter*, but in *Acinetobacter baumannii*, a TonB-dependent copper receptor leads to a dramatic decrease in biofilm formation (46). Among mutants with increased biofilm formation, CC_2264, a phosphomannomutase/phosphoglucomutase (PMM/PMG), shows particular promise as a novel biofilm factor. In *Pseudomonas aeruginosa*, AlgC acts as a bifunctional PMM/PMG to regulate exopolysaccharide production and specificity (47). The only polysaccharide known to be required for biofilm formation in *Caulobacter* is holdfast. It is thus tempting to speculate that PMM/PMG may act on holdfasts or that additional exopolysaccharides may be involved in biofilm formation. CC_2264 mutants also displayed increased adherence to cheesecloth (45), supporting the importance of this gene in biofilm development. Additional studies will be needed to determine the specific roles these new candidates play in *Caulobacter* biofilm development.

### Conclusion

Our study presents both a new resource for the community for holistically assessing *C. crescentus* physiology and new data on biofilm formation in this species. Our findings confirm previous results, thereby validating the library and our approach. We also find new mutants and regulatory interactions, raising new mechanistic questions for future studies that one would not have known to address otherwise. The CauloKO library is particularly promising for tackling new genetic screens that require clonal populations or could not otherwise be performed by TnSeq, such as the crystal violet screen presented in this study. Our work also presents a new sequencing analysis approach for decoding barcoded pools of transposon mutants. The CauloKO GUI, though used only for this study at present, allows for visual representation of sequencing data that is user-friendly and more intuitive than other sequencing pipelines and may thus be useful for other applications. We have thus made this GUI open source and available for the community. Finally, we hope that the online CauloKO portal will promote efforts across the *Caulobacter* community to make this library a dynamic and long-lasting resource to enable future discoveries.

## MATERIALS AND METHODS

### Bacterial strains and growth conditions

For this study, an overnight culture is defined as a single colony from petri dishes inoculated into 5 mL tubes and grown for 16 h. Exponential phase cultures were obtained by a 20-fold back dilution of overnight culture in fresh media and grown to an OD_660_ of ~0.5. *C. crescentus* laboratory strains (CB15) were grown in shaking culture at 30°C in PYE media, and pMiniHimar1 containing *E. coli* (WM3064) was grown as described previously (13, 48).

### Library preparation and sequencing

To generate mutant colonies, estimate the mutant population needed for saturation, and create sequencing pools for the CauloKO library, we utilized the methodology as described in the Knockout Sudoku pipeline with parental strain *C. crescentus* CB15 and pMiniHimar1 containing *E. coli* WM3064 (13). The only modification made to this protocol was selecting for mutant colonies on PYE plates containing kanamycin and nalidixic acid. Paired-end 150 nt Illumina MiSeq sequencing was performed on all sequencing pools at Princeton University’s Genomics Core and analyzed using Knockout Sudoku computational tools. Mapping of transposon insertion sites was made using the *C. crescentus* CB15 genome (GenBank accession no: AE005673). For determining the physical location of mutants, we generated a Matlab-based CauloKO GUI for determining sequencing read counts for all mutants in the collection. Functions of the GUI are described in greater detail in Fig. S2. We used an absolute read count threshold of 50 read counts for primary thresholding and an SNR of 9 for secondary thresholding. Sanger sequencing verification of mutants was accomplished through Genewiz.

### Crystal violet assay

Wild-type and CauloKO mutants were statically grown for 48 h at 30°C in 96-well plates to ensure saturation of growth. Absorbance (OD_660_) was measured using a plate reader to obtain the density of the culture, and then washed with deionized water. Crystal violet (0.5% wt/vol in 80% dH_2_0, 20% methanol) was added to the plates and incubated at room temperature for 10 min before washing again in deionized water. Crystal violet was resolubilized using 95% ethanol as a solvent and absorbance (OD_540_) was measured using a plate reader. Biofilm production was measured by normalizing the absorbance of the crystal violet to the absorbance of the bacterial growth (OD_540_/OD_660_). For the crystal violet screen, each consolidated CauloKO library plate was measured in biological triplicate. For targeted candidates in the collection, mutants were measured in biological triplicate with each experiment performed with four technical replicates. The wild-type used for experiments was the *C. crescentus* CB15 parental strain of the library.

## SUPPLEMENTAL MATERIAL

Supplemental figure and table legends jb.00417-22-s0001.docx Legends for Figures S1 and S2, and Tables S1 to S4.

Figure S1 jb.00417-22-s0002.pdf Monte Carlo simulation for number of Tn mutants needed for CauloKO library generation.

Figure S2 jb.00417-22-s0003.pdf CauloKO GUI layout and description of functions.

Table S1 jb.00417-22-s0004.xlsx Table S1.

Table S2 jb.00417-22-s0005.xlsx Results indicating that 40 of the 53 mutants (75%) contained insertions in either the predicted gene or the gene immediately adjacent.

Table S3 jb.00417-22-s0006.xlsx Complete collection.

Table S4 jb.00417-22-s0007.xlsx Consolidated collection of the library containing 2902 mutants with a single mutant for each available ORF.

ASM does not own the copyrights to Supplemental Material that may be linked to, or accessed through, an article. The authors have granted ASM a non-exclusive, world-wide license to publish the Supplemental Material files. Please contact the corresponding author directly for reuse.
