# Supplementary material for: CauloKO: an ordered transposon mutant library in Caulobacter crescentus
Source: J Bacteriol. 2026 Feb 27;208(3):e00417-22. doi: 10.1128/jb.00417-22 (PMC13001252; doi:10.1128/jb.00417-22)
Supplement: Supplemental figure and table legends — Legends for Figures S1 and S2, and Tables S1 to S4. [file jb.00417-22-s0001.docx]

**Supplementary Figure 1: Mutants required for saturation of ORFs as determined by Knockout Sudoku pipeline.** Solid black line represents the total number of ORFs in the *C. crescentus* CB15 genome. Dashed line represents the number of mutants picked for library construction.

**Supplemental Figure 2: CauloKO GUI for sequence data analysis.**

**Supplemental Table 1: SNPs present in parental strain and transposon mutant from library collection.** SNPs have been aligned by row based on their similarity to the parental *C. crescentus* CB15 strain. Green indicates shared sequence similarity between the parental strain and at least one transposon mutant.

**Supplementary Table 2: Identity of transposon mutants by Sanger Sequencing.** Green indicates matches between Sanger results and library annotation. Yellow indicates partial matching most likely due to arbitrary PCR amplification schema. Red indicates contradiction between Sanger results and library annotation

**Supplemental Table 3: Complete CauloKO Library Collection.**

**Supplemental Table 4: Consolidated CauloKO Library Collection.**
