# Supplementary figures and images for: CauloKO: an ordered transposon mutant library in Caulobacter crescentus
Source: J Bacteriol. 2026 Feb 27;208(3):e00417-22. doi: 10.1128/jb.00417-22 (PMC13001252; doi:10.1128/jb.00417-22)

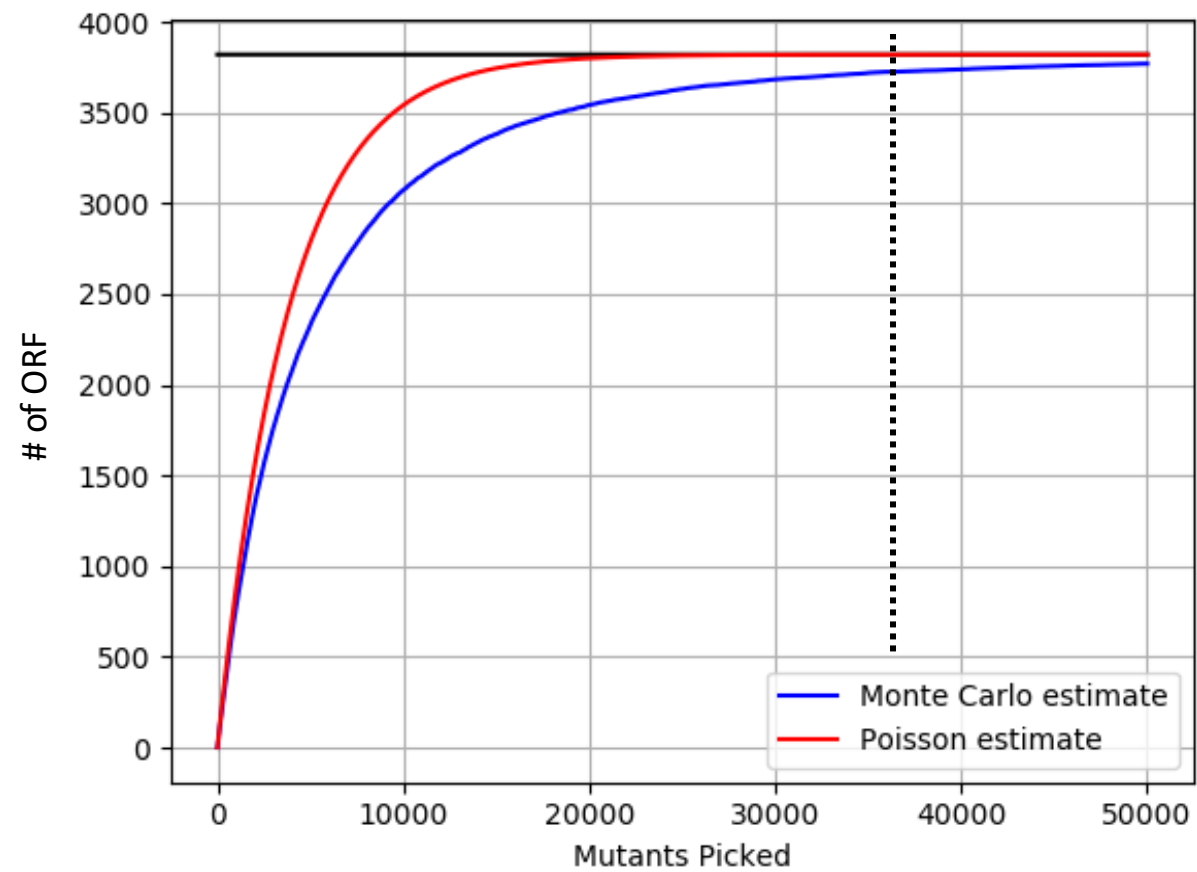

Supplement: Figure S1 — Monte Carlo simulation for number of Tn mutants needed for CauloKO library generation. [file jb.00417-22-s0002.pdf]
