# Supplementary material for: CauloKO: an ordered transposon mutant library in Caulobacter crescentus
Source: J Bacteriol. 2026 Feb 27;208(3):e00417-22. doi: 10.1128/jb.00417-22 (PMC13001252; doi:10.1128/jb.00417-22)
Supplement: Figure S2 — CauloKO GUI layout and description of functions. [file jb.00417-22-s0003.pdf]

Supplementary Figure 2: CauloKO GUI for coordinate determination of mutants.

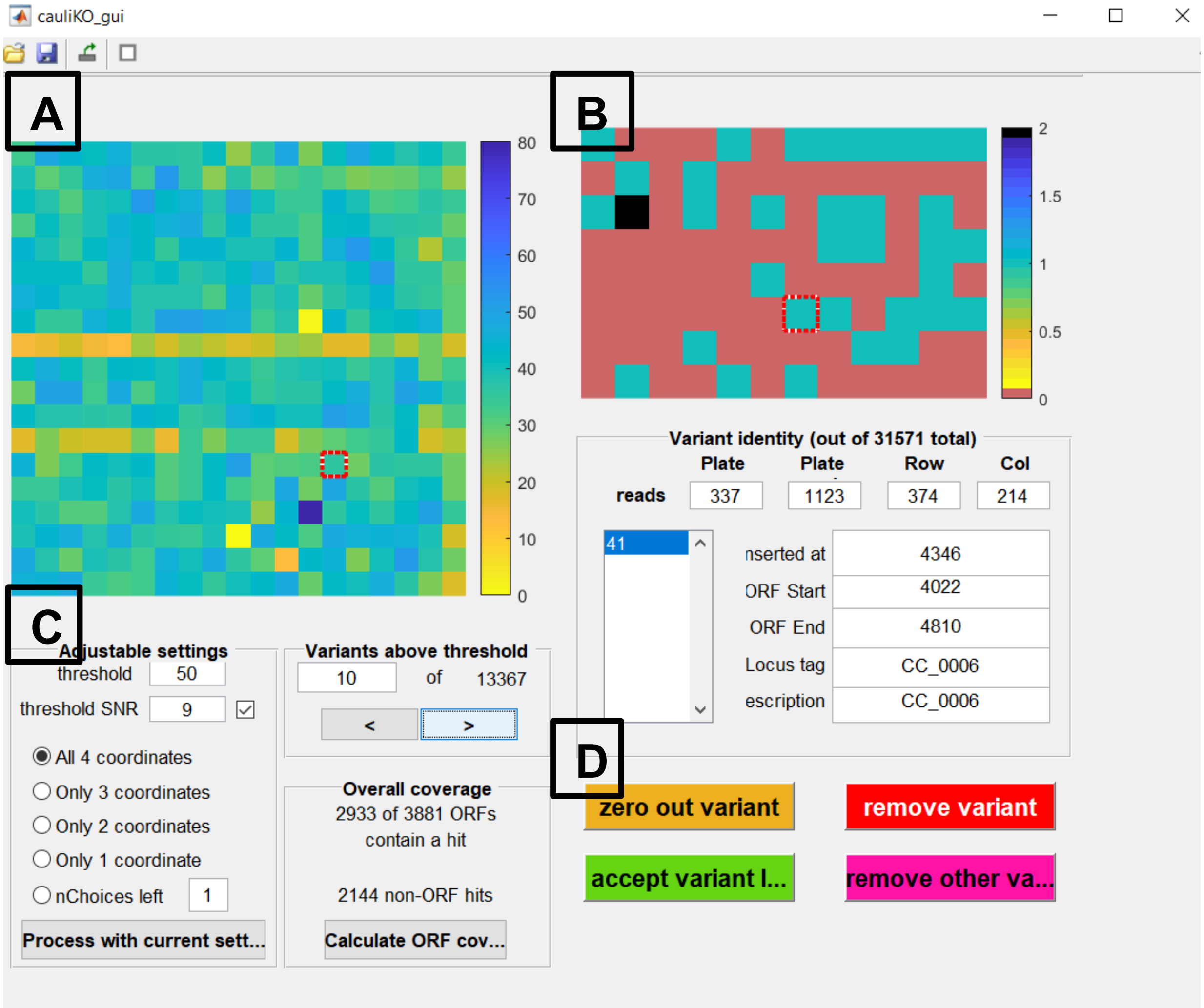

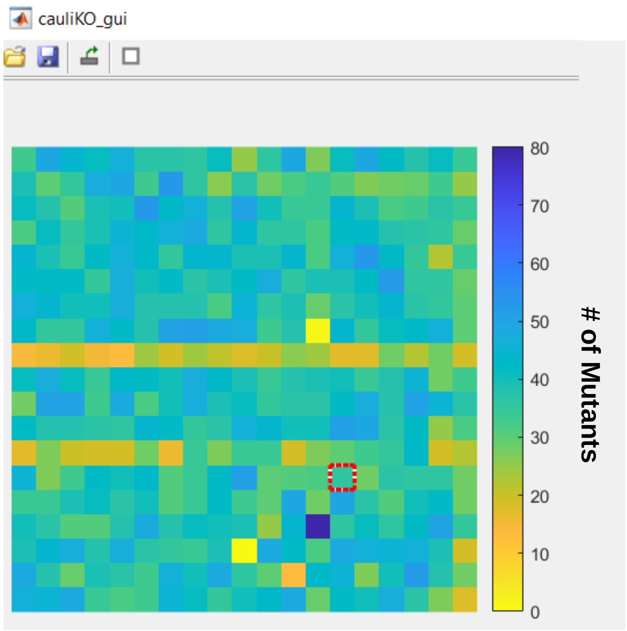

A. Heat-map displaying number of mutants with identifiable location coordinates in 19 x 19 plate grid. Red square denotes current 96-well plate selected (ex: 218).

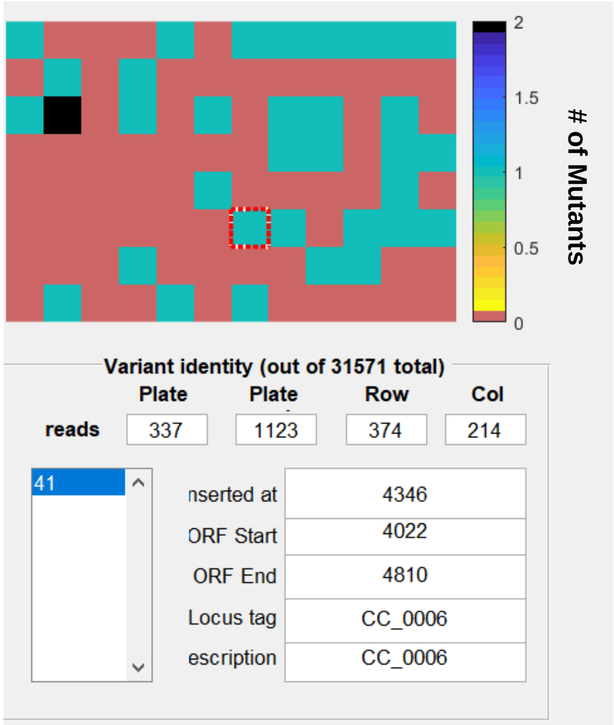

B. Heat-map displaying number of mutants with identifiable location coordinates in 96-well plate. Red square denotes current well (ex: F7). Variant identity panel lists transposon mutants with possible locations in the given well. Sequencing read counts for the plate column, plate row, row, and column coordinates are also given.

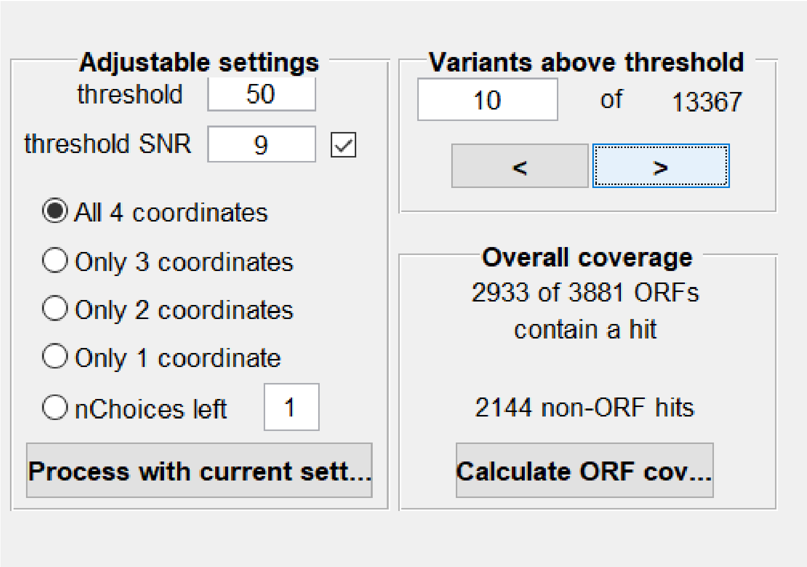

C. Determining mutant locations based on sequencing thresholds. “Threshold” uses the absolute number of read counts for a given coordinate as a cutoff for location identity. “Threshold signal-to-noise ratio (SNR)” uses the ratio of the highest read counts to the second highest read counts for a given coordinate (i.e, plate row, plate column, row, or column) as a cutoff for identity location. Check box allows for one or both of these cutoffs to be used in the analysis. Options give lists of mutants for which 1 to 4 of the coordinates are known or mutants with have n number of possible locations. Variant in list is depicted in the top right corner with overall ORF coverage given in the bottom right corner.

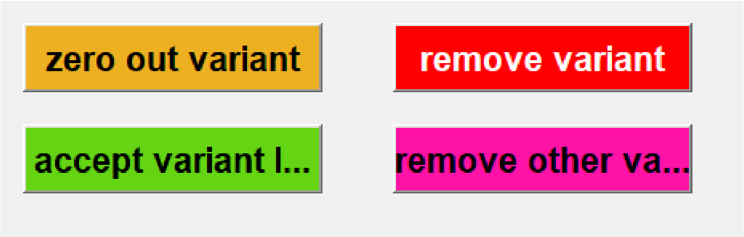

D. Options for editing sequencing data for a particular mutant. “Zero out” creates 0s for all read counts for a selected mutant, making it below the threshold selected. “Accept” creates 0s for all read count for all coordinates except the current location. “Remove” removes the selected mutant from the data. “Remove other” removes all other variants from a particular well selected.
